# Non‐m^6^A RNA modifications in haematological malignancies

**DOI:** 10.1002/ctm2.1666

**Published:** 2024-06-16

**Authors:** Meiling Chen, Yuanzhong Chen, Kitty Wang, Xiaolan Deng, Jianjun Chen

**Affiliations:** ^1^ Department of Hematology Fujian Institute of Hematology Fujian Provincial Key Laboratory on Hematology Fujian Medical University Union Hospital Fuzhou China; ^2^ Department of Systems Biology Beckman Research Institute of City of Hope Monrovia California USA; ^3^ Gehr Family Center for Leukemia Research City of Hope Medical Center and Comprehensive Cancer Center Duarte California USA

**Keywords:** epitranscriptomics, haematological malignancies, non‐m^6^A RNA modification

## Abstract

Dysregulated RNA modifications, stemming from the aberrant expression and/or malfunction of RNA modification regulators operating through various pathways, play pivotal roles in driving the progression of haematological malignancies. Among RNA modifications, *N* ^6^‐methyladenosine (m^6^A) RNA modification, the most abundant internal mRNA modification, stands out as the most extensively studied modification. This prominence underscores the crucial role of the layer of epitranscriptomic regulation in controlling haematopoietic cell fate and therefore the development of haematological malignancies. Additionally, other RNA modifications (non‐m^6^A RNA modifications) have gained increasing attention for their essential roles in haematological malignancies. Although the roles of the m^6^A modification machinery in haematopoietic malignancies have been well reviewed thus far, such reviews are lacking for non‐m^6^A RNA modifications. In this review, we mainly focus on the roles and implications of non‐m^6^A RNA modifications, including *N* ^4^‐acetylcytidine, pseudouridylation, 5‐methylcytosine, adenosine to inosine editing, 2′‐O‐methylation, *N* ^1^‐methyladenosine and *N* ^7^‐methylguanosine in haematopoietic malignancies. We summarise the regulatory enzymes and cellular functions of non‐m^6^A RNA modifications, followed by the discussions of the recent studies on the biological roles and underlying mechanisms of non‐m^6^A RNA modifications in haematological malignancies. We also highlight the potential of therapeutically targeting dysregulated non‐m^6^A modifiers in blood cancer.

## Graphical abstract

1. Non‐m^6^A RNA modifications play critical roles in haematological malignancies.
2. Gaps exist in understanding of non‐m^6^A modifications and their regulatory mechanisms in blood cancers.
3. Emerging studies show non‐m^6^A modification regulators as potential prognostic markers and therapeutic targets in blood malignancies.
4. Developing specific inhibitors for non‐m^6^A RNA modifications in clinical settings is still in its infancy.

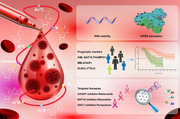

## 1 INTRODUCTION

**FIGURE 1:**
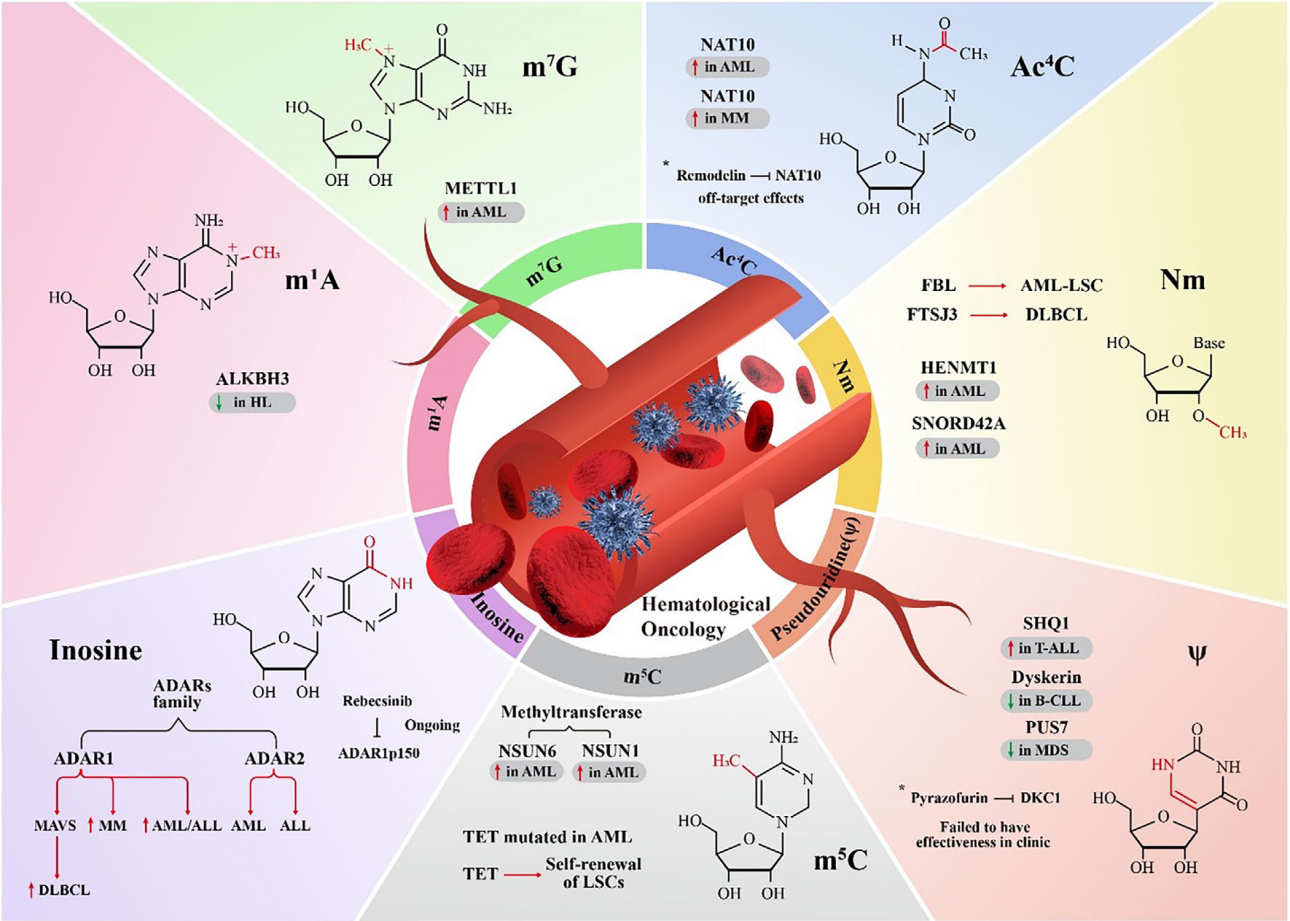
Major non‐*N* ^6^‐methyladenosine (m^6^A) RNA modification regulators in the progression of different haematological oncologies. Non‐m^6^A RNA modifications such as *N* ^4^‐acetylcytidine (Ac^4^C), pseudouridylation (Ψ), 5‐methylcytosine (m^5^C), inosine, 2′‐O‐methylation (N_m_), *N* ^1^‐methyladenosine (m^1^A) and *N* ^7^‐methylguanosine (m^7^G) are linked to specific enzymes and blood malignancies. Methyltransferase 1 (METTL1) is increased in acute myeloid leukaemia (AML), ALKBH3 is reduced in Hodgkin lymphoma (HL) and N‐acetyltransferase 10 (NAT10) is elevated in both AML and multiple myeloma (MM). The roles of FTSJ3 in DLBCL, HENMT1 in AML and the promoting effect of fibrillarin (FBL) on AML‐LSC are detailed, along with the involvement of SNORD42A in AML. The activity of the ADAR family in AML, acute lymphoblastic leukaemia (ALL), MM and DLBCL; the influence of ten‐eleven translocation (TET) mutations on leukaemia stem cell (LSC) self‐renewal in AML, and the role of NSUN6 and NSUN1 in AML are illustrated. Also depicted are the elevation of SHQ1 in T‐acute lymphoblastic leukaemia (T‐ALL), the decline of dyskerin in B‐chronic lymphocytic leukaemia (B‐CLL) and the reduction of pseudouridine synthase 7 (PUS7) in myelodysplastic syndrome (MDS). Therapeutic perspectives with drugs such as Remodelin, Rebecsinib and Pyrazofurin are also depicted, showcasing their clinical outcomes. DLBCL, Diffuse Large B‐Cell Lymphoma.

The activities of biological macromolecules are controlled in a precise and effective manner by chemical alterations.^1^ Proteins, RNA, DNA, carbohydrates and lipids are among the biological macromolecules that may undergo post‐synthesis modifications.^1^ In this regard, RNA has been shown to undergo more modifications compared to those of other macromolecules.^1^ The term ‘epitranscriptomics’ has been used to describe the chemical alterations of RNA that control gene expression irrespective of RNA sequence.^1^, ^2^, ^3^, ^4^ To date, more than 170 different RNA modifications have been discovered in coding and non‐coding RNAs (ncRNAs).^3^, ^5^ *N* ^6^‐methyladenosine (m^6^A), *N* ^4^‐acetylcytidine (Ac^4^C), pseudouridylation (Ψ), 5‐methylcytosine (m^5^C), adenosine to inosine (A‐to‐I) editing, 2′‐O‐methylation (N_m_), *N* ^1^‐methyladenosine (m^1^A) and *N* ^7^‐methylguanosine (m^7^G) are among the most prevalent RNA modifications in eukaryotes.^6^ Studies indicate that RNA modifications modulate the chemical, physical and topological characteristics of RNA, but the specific functions of many RNA modifications remain unknown. Methylation stands out as the predominant form of RNA modification, constituting a substantial 66% of all identified RNA modifications.^7^ The therapeutic potential and the yet‐to‐be‐discovered biological natures of such modifications fuel interest in this field. Growing evidence has demonstrated that the pathophysiology of human diseases, including different types of malignancies, involves the dysregulation of RNA modifications.^8^ Writers, readers and erasers are the three classes of RNA modification regulators, and dysregulation in each class may contribute to the development of tumours.^9^, ^10^ Recently, the functions of RNA modification regulators, especially m^6^A regulators, in the progression of haematological malignancies and their potential as therapeutic targets have been the subjects of extensive investigations.^11^, ^12^, ^13^, ^14^, ^15^, ^16^, ^17^, ^18^, ^19^, ^20^, ^21^, ^22^, ^23^, ^24^, ^25^, ^26^, ^27^ We and others have demonstrated that RNA m^6^A writers (methyltransferases, such as METTL3, METTL14 and METTL16), erasers (demethylases, including FTO and ALKBH5) and readers (RNA‐binding proteins that specifically recognise m^6^A modification, including YTHDF1/2/3, YTHDC1/2 and IGF2BP1/2/3) are frequently dysregulated in various types of cancers and play essential oncogenic or tumour‐suppressor roles in cancer initiation, progression, maintenance, metabolism, metastasis, drug response and immune evasion, as well as in cancer stem cell self‐renewal; the dysregulated m^6^A regulators appears to be promising therapeutic targets for the treatment of blood cancer.^1^, ^3^, ^4^, ^11^, ^12^, ^13^, ^14^, ^15^, ^16^, ^17^, ^18^, ^19^, ^20^, ^22^, ^23^, ^24^, ^28^, ^29^, ^30^ Because the functions and therapeutic implications of m^6^A modification in haematopoietic malignancies have been well reviewed elsewhere,^1^, ^3^, ^4^, ^28^, ^29^, ^30^ this review centers its attention on non‐m^6^A RNA modifications.^31^ In this review, we provide a concise yet comprehensive overview of the latest research on different non‐m^6^A RNA modifications, regulators and roles in blood malignancies (Figure 1).

## 2 CELLULAR FUNCTIONS AND REGULATORY ENZYMES OF NON‐m^6^A RNA MODIFICATIONS

### 2.1 Ac^4^C

As the first acetylating decoration, Ac^4^C modification plays a crucial role in gene expression regulation.^32^ Studies of Ac^4^C were initially conducted on yeast and bacterial tRNA in 1966,^33^ and then expanded to eukaryotic t‐ and 18S r‐RNAs.^32^ Ac^4^C exists on tRNAs, which increases the accuracy of protein translation and keeps the organism's thermotolerance intact.^32^ For thermophilic organisms, Ac^4^C on rRNAs is pivotal in safeguarding translation fidelity.^32^, ^34^ In 2018, Arango et al. first reported antibody‐based mapping evidence for the Ac^4^C modification on mRNAs, which is positively correlated to the lifetime of mRNAs, with functions on the enhanced translation efficiency and the regulation of gene expression by the existence in the wobble regions of cytidine (at coding region of mRNA).^35^ The reported proportion of Ac^4^C in mammalian polyadenylated RNA ranges from .01% to .36%.^31^, ^36^ Recently, the same group delineated that Ac^4^C modifications at the 5′‐untranslated region (UTR) region potentially form the barrier for ribosome scanning, thus inhibiting translation initiation activites.^37^ Regrettably, the absence of base‐resolution quantification for individual Ac^4^C sites has hindered the possibility of orthogonal validation and functional prioritisation based on modification stoichiometries.^38^ Therefore, the quantitative distributions of Ac^4^C among rRNA, tRNA and mRNA have yet to be comprehensively defined in any organism.

**FIGURE 2:**
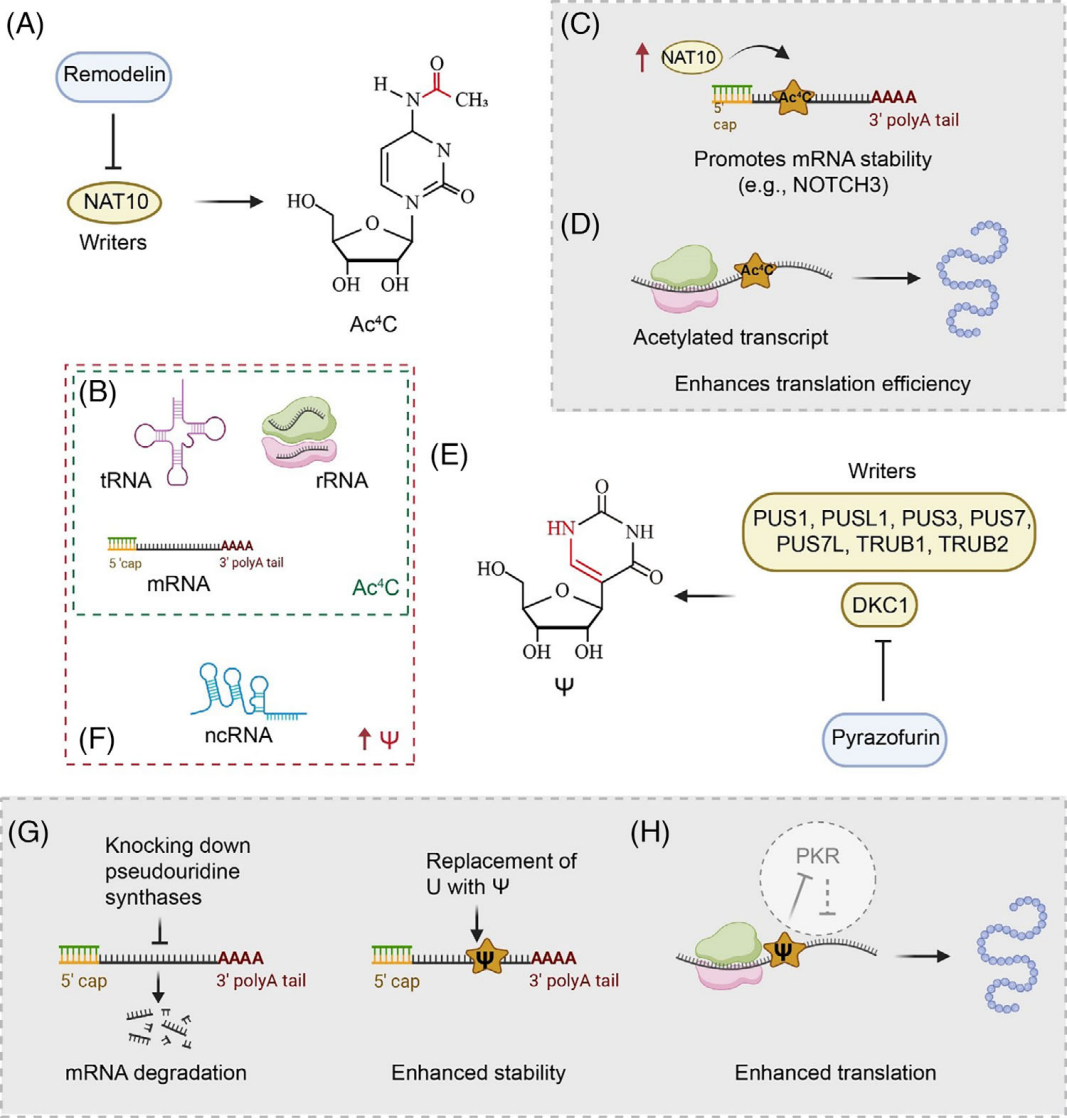
The regulation of *N* ^4^‐acetylcytidine (Ac^4^C) and pseudouridylation (Ψ) in transcription and translation. (A and B) The role of N‐acetyltransferase 10 (NAT10) in catalysing Ac^4^C modification, targeted and inhibited by Remodelin (A) across tRNA, rRNA and mRNA (B). (C) Ac^4^C modification‐mediated stabilisation of target mRNAs in NAT10‐overexpressing cells. (D) Ac^4^C enhancement of translation efficiency, extending beyond the initiation phase. (E) Pseudouridylation of mRNA, converting uridine to Ψ, is catalysed by pseudouridine synthase 1 (PUS1), PUSL1, PUS3, PUS7, PUS7L, TRUB pseudouridine synthase family member 1 (TRUB1), TRUB2 and dyskerin pseudouridine synthase 1 (DKC1). Pyrazofurin (an inhibitor of DKC1) can impede this process. (F) Elevated levels of Ψ in cellular non‐coding RNAs. (G) Ψ enhances mRNA structural stability, actively protecting target mRNAs from degradation pathways. (H) Ψ enhances translation by reducing RNA‐dependent protein kinase (PKR) activation in RNA.

N‐acetyltransferase 10 (NAT10) is a ‘writer’ of Ac^4^C that catalyses the production of Ac^4^C RNA modification of tRNA, rRNA and mRNA, and requires acetyl‐coenzyme A (CoA) to provide the acetyl group and ATP/guanosine triphosphate (GTP) hydrolysis to provide energy^39^ (Figure 2A,B). Two helpers, THUMP domain containing 1 (THUMPD1) and the antisense sequence of small nucleolar RNA (snoRNA), facilitate NAT10 binding to the target sequences in the Ac^4^C modification region of tRNA and 18S rRNA, respectively.^40^

Ac^4^C exerts a significant influence on RNA metabolism, affecting both the stability and translation efficiency. Liao et al. reported that Ac^4^C modifications lead to increased stability of target mRNAs, such as *NOTCH3*, evident from the prolonged half‐lives in NAT10‐overexpressing cells^41^ (Figure 2C). The localisation of Ac^4^C near translation start sites within coding sequences, as revealed by acetylated RNA immunoprecipitation and sequencing (acRIP‐seq), is a key in mRNA stability regulation and subsequent protein production.^41^ Furthermore, Ac^4^C boosts protein translation efficiency, with effects that extended beyond translation initiation, as demonstrated by 5′‐bromo‐uridine immunoprecipitation chase‐deep sequencing^35^ (Figure 2D).

### 2.2 Ψ

Ψ is one of the most prevalent post‐transcriptional RNA modifications and is found in multiple RNA species.^31^, ^42^ A group of highly conserved enzymes known as pseudouridine synthases (PUSs) facilitate the formation of Ψ, and 13 PUS members have been identified in eukaryotes thus far. Recent studies identified writers responsible for pseudouridylation of mRNAs, including PUS1, PUSL1, PUS3, PUS7, PUS7L, TRUB pseudouridine synthase family member 1 (TRUB1), TRUB2 and dyskerin pseudouridine synthase 1 (DKC1)^43^, ^44^ (Figure 2E). The Ψ/U ratio has been quantified to be approximately .2–.6% in mRNA^45^, ^46^ and 1.4% in rRNA.^42^ These enzymes are responsible for depositing Ψ modification onto RNA molecules, either independently or by engaging in an RNA‐dependent process that includes a diverse array of antisense box H/ACA snoRNAs. Cellular ncRNAs contain a higher amount of Ψ, a C5‐glycoside isomer of uridine, than any other post‐transcriptional modifications^47^ (Figure 2F). Pseudouridylated rRNAs and small nuclear RNAs (snRNAs) are necessary for the proper performance of the ribosome and spliceosome.^48^ In eukaryotes, a family of box H/ACA small nucleolar ribonucleoproteins (snoRNPs), which includes the core proteins Cbf5, Nhp2, Nop10 and Gar1, mostly governs pseudouridylation.^49^ The RNA component acts as a base‐pairing guide to direct the Cbf5 enzyme (called dyskerin in humans) towards a specific location to initiate the pseudouridylation process.^50^ Before dyskerin protein complex assembly with H/ACA snoRNA, SHQ1 acts as an assembly chaperone to prevent complex aggregation due to non‐specific RNA binding.^51^ The stabilisation of RNA conformations and the destabilisation of various RNA‐binding proteins are some of the less known molecular activities of pseudouridine, raising the possibility that RNA pseudouridylation may have a broader range of impacts on RNA metabolism and tumourigenesis than currently appreciated.^52^

Ψ significantly enhances RNA stability, as demonstrated by reduced target mRNA levels in yeast when PUSs are knocked out.^53^ This decrease in target mRNA levels indicates that Ψ plays a role in increasing mRNA structural stability, potentially by extending mRNA lifetime through spatially sequestering it from degradation pathways. Experiments demonstrated that the non‐canonical base pairing of Ψ enhanced RNA stability compared to that of uridine,^54^, ^55^ highlighting its pivotal role in influencing the susceptibility of RNA to degradation (Figure 2G). Ψ also influences translation initiation, especially in the innate immune response, by reducing RNA‐dependent protein kinase (PKR) activation in RNA, likely due to changes in RNA secondary structure^56^, ^57^ (Figure 2H). This modification also helps distinguish self from non‐self RNA, which is crucial for preserving normal translation processes during immune responses.^56^, ^57^ Ψ located in stop codons of mRNAs can cause partial readthrough in vitro and in vivo.^43^, ^58^, ^59^

### 2.3 m^5^C

**FIGURE 3:**
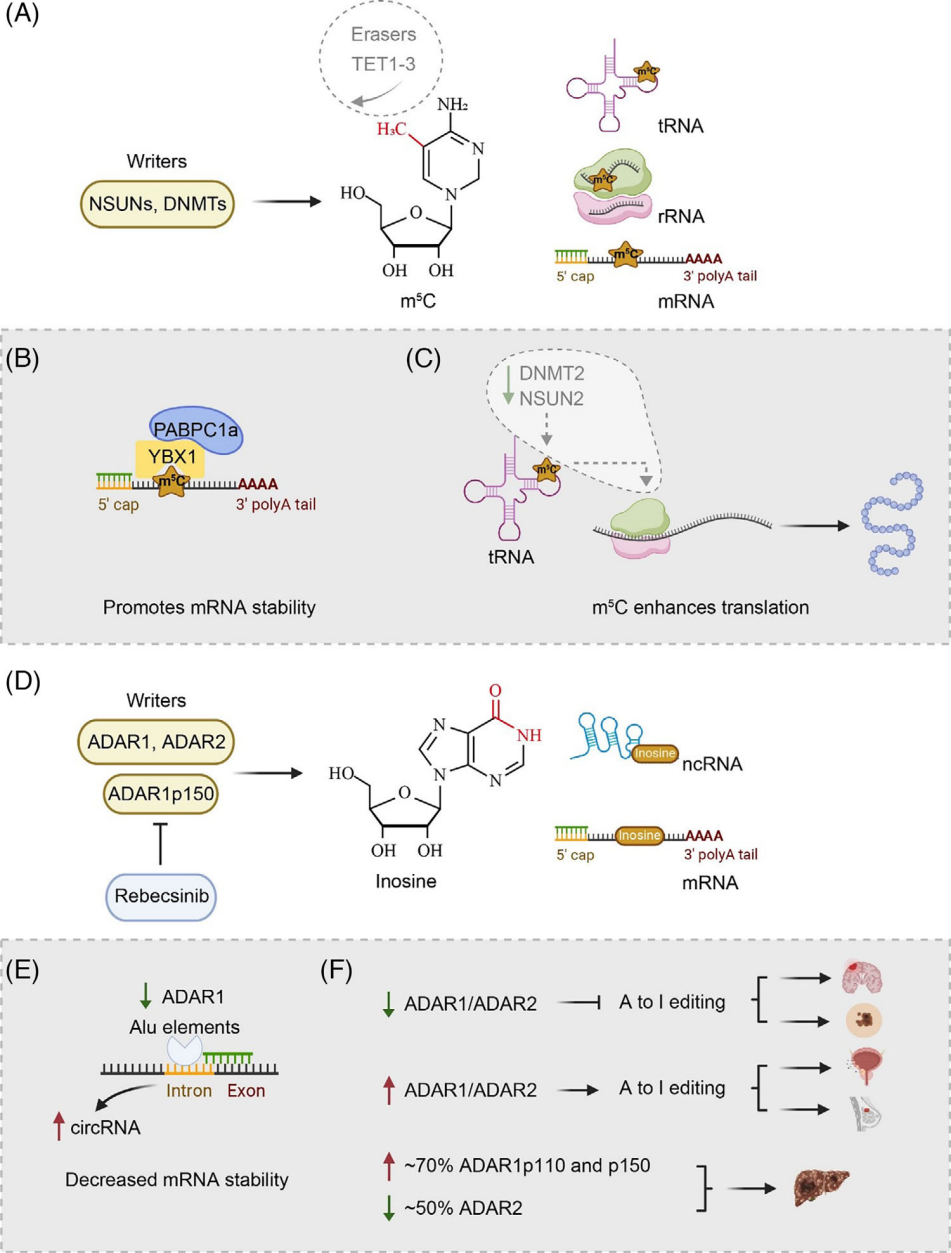
Modulation of gene expression by 5‐methylcytosine (m^5^C) and the influence of inosine on cancer development. (A) The regulation of m^5^C through catalysis by the NSUNs family and DNA methyltransferases (DNMTs), with demethylation mediated by ten‐eleven translocation (TET)1‐3. (B) YBX1 and PABPC1a specifically recognise m^5^C‐modified mRNAs to maintain mRNA stability. (C) m^5^C modification in tRNA (DNMT2/NSUN2‐mediated methylation) enhances translation efficiency. (D) Adenosine to inosine (A‐to‐I) editing is primarily regulated by ADAR1/ADAR2, with Rebcesinib inhibiting ADAR1p150. (E) Decreased ADAR1 in intronic Alu elements leading to higher circRNA levels and lower mRNA stability. (F) The varied expression patterns of ADAR1/ADAR2 across different cancers: reduced expression in brain tumours and melanomas (top), upregulated in prostate and breast cancers (middle) and increased ADAR1 but decreased ADAR2 expression in liver cancer (bottom).

The first m^5^C modification of eukaryotic mRNA was discovered in the 1970s, and there is emerging evidence that m^5^C is a significant epitranscriptomic signature of RNA.^60^, ^61^ m^5^C, found in tRNAs, rRNAs and most recently, poly(A)RNAs, has received relatively little attention.^62^ Specially, the abundance proportion of m^5^C/C modifications in human mRNA is approximately between .02% and .09%.^63^, ^64^ The methylation of the carbon 5 position in cytosine (m^5^C) within RNA molecules is a biologically significant process catalysed by ‘writers’, NOL1/NOP2/SUN domain (NSUN) family of methyltransferases, such as NSUN1‐7,^65^ or DNA methyltransferases (DNMT1‐3).^66^ m^5^C can undergo oxidative modification via ten‐eleven translocation (TET) family demethylases, including TET1‐3, as ‘erasers’, leading to the generation of 5‐hydroxymethylcytosine and various other oxidative metabolites^30^ (Figure 3A). Among its functions, this modification is added to several RNA classes by RNA methyltransferases that are localised in diverse parts of the cell.^60^ These include mRNAs and other ncRNAs besides cytoplasmic and mitochondrial rRNAs and tRNAs. Accordingly, m^5^C modifications may have a variety of methods to impact RNA metabolism due to numerous potential targets.^67^ RNAs can be labelled with m^5^C modification through a complex of proteins in *Homo sapiens*, including NSUN1‐7 and DNMT2.^67^ The function of m^5^C residues in tRNAs and rRNAs during mRNA decoding on the ribosome remains poorly understood.

It has been discovered that m^5^C not only governs the stability of mRNA, but also plays a crucial role in rRNA and tRNA stability regulation. For example, Y‐box‐binding protein 1 (YBX1) recognises m^5^C in oncogenic transcripts and contributes to transcript stability through recruitment of embryonic lethal abnormal vision like 1 (ELAVL1) during both human tumourigenesis and metastasis.^68^ Moreover, YBX1 and its partner poly(A) binding protein cytoplasmic 1a (PABPC1a) were reported to specifically recognise and interact with m^5^C‐modified mRNAs and maintain maternal mRNA stability during the maternal‐to‐zygotic transition^69^ (Figure 3B). Furthermore, it was reported that m^5^C modifications in tRNA and rRNA play crucial roles in the translation regulation. For instance, methylation of tRNAAsp/Gly/Val at position C38 in the bone marrow (BM) by DNMT2 can influence specific protein synthesis^70^ (Figure 3C). Conversely, the function of m^5^C residues in tRNAs and rRNAs during mRNA decoding on the ribosome remains poorly understood.

### 2.4 A‐to‐I editing

The conversion of A‐to‐I in RNA, catalysed by adenosine deaminase (ADAR), is crucial for a wide range of biological functions and has been linked to multiple disorders.^71^ Therefore, understanding RNA A‐to‐I editing sites is essential for both fundamental research and therapeutic developments. RNA A‐to‐I editing is mainly mediated by the adenosine deaminases acting on RNA (ADARs) family, namely, ADAR1 and ADAR2.^72^, ^73^ ADAR1 and ADAR2, functioning as ‘writers’ of A‐to‐I editing, demonstrate a marked preference for deaminating adenosines within double‐stranded RNA (dsRNA) regions (Figure 3D). Given that inosine preferentially base pairs with cytidine, any inosine‐based mismatches can lead to significant effects on various essential cellular processes. For instance, A‐to‐I editing of both host and viral transcripts has been observed in Kaposi's sarcoma‐associated herpesvirus (KSHV)‐infected cells.^74^ Additionally, this A‐to‐I editing pathway undergoes further expansion during KSHV lytic reactivation, effectively preventing recognition and detection by RIG‐I‐like receptors (RLRs) pathway. Furthermore, ADAR2 edits its own pre‐mRNA and creates an alternative splicing acceptor site, effectively leading to the suppression of its own expression.^75^ In addition, inosine has been reported as a modulator of mRNA stability and expression through influencing interactions between microRNAs (miRNAs) and mRNAs.^76^ In addition to its effects on untranslated regions, A‐to‐I editing can also take place in the translated regions of mRNA, potentially introducing amino acid substitutions that alter protein function.^77^ Due to the properties of A‐to‐I editing, ADARs represent potential therapeutic targets for multiple diseases.

A‐to‐I RNA editing crucially influences RNA stability and translation. This form of editing, occurring primarily within Alu elements in introns, is notably reduced in heart failure, leading to an increase in circRNA levels and a corresponding decrease in RNA stability^78^, ^79^ (Figure 3E). As observed in cancers^80^, ^81^ as well as in neurological^82^ and cardiac diseases,^83^ reduction in A‐to‐I editing indirectly affects the translation process, indicating its significance in gene regulation and disease progression^79^ (Figure 3F). A‐to‐I editing modulates steady‐state transcript levels by decreasing RNA stability, as demonstrated in the dihydrofolate reductase (DHFR) example, where miR‐125a‐3p links an editing quantitative trait loci (edQTL) with the DHFR expression quantitative trait loci (eQTL) and reduces the stability of unedited transcripts, leading to altered gene expression.^84^, ^85^ Additionally, A‐to‐I editing also impacts translation by generating eQTL signals from edQTLs through miRNA‐mediated transcript degradation.^85^

### 2.5 N_m_

**FIGURE 4:**
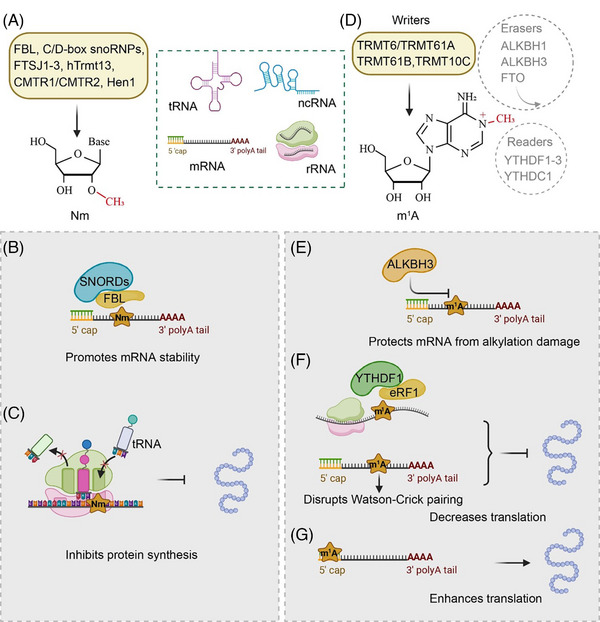
Roles of 2′‐O‐methylation (N_m_) and *N* ^1^‐methyladenosine (m^1^A) modifications in mRNA stability and translation processes. (A) Fibrillarin (FBL), small nucleolar ribonucleoproteins (snoRNPs), FTSJ1‐3, hTrmt13, CMTR1/CMTR2 and Hen1 mediate N_m_ modification in tRNA, mRNA, rRNA and non‐coding RNA (ncRNA). (B) FBL and SNORDs play a crucial role in promoting the stability of mRNA. (C) N_m_ modification within coding regions interferes with tRNA decoding during elongation, impeding the translation process. (D) m^1^A modification is installed by TRMT6/TRMT61A, TRMT61B and TRMT10C. Demethylation of m^1^A is catalysed by ALKBH1, ALKBH3 and FTO. m^1^A is recognised by YTHDF1‐3 and YTHDC1. (E) ALBKH3 protects mRNA from degradation. (F and G) m^1^A exhibits dual effects on mRNA translation. m^1^A recruits YTHDF1 and eRF1, or disrupts Watson‐Crick pairing in mitochondrial RNA, inhibiting translation efficiency (F). m^1^A located at the 5′ cap enhances translation (G).

N_m_ is one of the most frequent RNA modifications and involves the replacement of a hydrogen atom (‒H) at the 2ʹ‐hydroxyl (‒OH) position of the ribose sugar with a methyl group (‒CH_3_). This RNA modification impacts RNA molecules in a variety of ways via alterations in structure, stability and interactions.^86^ It also affects a range of cellular functions, such as self‐recognition, non‐self‐recognition and epitranscriptomic gene regulation.^87^ Drazkowska et al. demonstrated that N_m_ of the mRNA 5ʹ cap in mammals plays pivotal roles in distinguishing ‘self’ from ‘non‐self’ during viral infections, influencing protein production in a cell‐specific manner and contributing to a transcript's resistance against host innate immune response and decapping exoribonuclease (DXO)‐mediated decapping and degradation.^88^ Recent developments have shown that N_m_ sites are also found on RNAs (tRNA, rRNA, mRNA and ncRNA) and their presence is linked to a variety of disorders.^86^ Dai et al. first determined the total N_m_ levels in human mRNA and found that the N_m_/N molar ratios ranged from .012% for A_m_/A to .15% for U_m_/U.^89^ N_m_ modification of most rRNAs and snRNAs is primarily catalysed by C/D‐box snoRNPs, for instance, SNORD42A.^90^, ^91^ FTSJ1/2/3 has been shown to be a human tRNA 2′‐O‐methyltransferase.^92^, ^93^ FTSJ1‐mediated N_m_ modification of tRNA plays a pivotal role in substantially reducing the expression of DRAM and thereby inhibiting the progression of non‐small cell lung cancer.^94^ Recently, Li et al. demonstrated that hTrmt13 is a 2′‐O‐methyltransferase at position 4 of tRNA and may enhance translation by suppression of tRNA‐derived small fragments (tRFs).^95^ In addition, Cap methyltransferase 1 (CMTR1) and CMTR2 were both identified as mRNA 2′‐O‐methyltransferases,^96^ and Hua enhancer 1 (Hen1), a small RNA 2′‐O‐methyltransferase, was found to be dysregulated in different cancer cells.^97^ Notably, Hua enhancer 1 could protect miRNA from exonuclease cleavage, strengthens their binding to AGO2, enhances piwi‐interacting RNA (piRNA) stability and contributes to the maintenance of piRNA length and abundance^98^, ^99^ (Figure 4A).

N_m_ plays a crucial role in RNA metabolism, impacting both RNA stability and translation through its interaction with various molecular pathways. In eukaryotic mRNAs, N_m_ modifications, often occurring at transcription start sites and within internal regions, are mediated by enzymes such as tRNA 2′‐O‐methyltransferases and fibrillarin (FBL), and are guided by C/D box snoRNAs.^100^ These modifications stabilise mRNA (Figure 4B). However, in translation, N_m_ modifications within coding regions disrupt tRNA decoding during elongation, inhibiting the translation process^101^ (Figure 4C). Additionally, presence of N_m_ near splice sites suggests its involvement in splicing events, further influencing mRNA processing and translation.^89^ This comprehensive role of N_m_ in mRNA stability, translation and potentially splicing underlines its significance in gene expression regulation.

### 2.6 m^1^A

In 1961, m^1^A was initially recognised as a universally preserved modification in tRNA and rRNA.^31^ In contrast to other more prevalent RNA modifications, m^1^A methylation adds a methyl ester to the nitrogen atom at the adenine 1 position of RNA molecules.^102^ The m^1^A methylation is largely abundant in the mRNA 5ʹ‐UTR, accounting for .015%−.054% in mammalian cells and about .16% in tissues.^103^, ^104^, ^105^ ncRNAs undergo m^1^A methylation to retain their structure and functionality.^106^ TRMT10C, TRMT61B and TRMT6/61A function as writers of m^1^A modification.^107^, ^108^ Demethylation of m^1^A can be catalysed by AlkB family enzymes, such as ALKBH1, ALKBH3^104^, ^105^, ^109^ and FTO.^110^ Studies have indicated that ALKBH3 is likely responsible for the demethylation of m^1^A in both mRNA and tRNA.^105^ In contrast, ALKBH1 and FTO have been observed to primarily demethylate m^1^A in tRNA, potentially impacting mRNA translation.^109^, ^110^ Moreover, it was reported that m^6^A reader proteins, YTH domain‐containing family protein 1 (YTHDF1), YTHDF2, YTHDF3 and YTH domain‐containing protein 1 (YTHDC1) read m^1^A methylation modification information, identifying and adhering to m^1^A methylation sites^111^, ^112^ (Figure 4D).

m^1^A regulated the stability of mRNAs, such as *CSF‐1*, where m^1^A modification influenced mRNA degradation, with the involvement of demethylase ALKBH3 altering stability^113^ (Figure 4E). Meanwhile, m^1^A can both inhibit and promote translation. For example, m^1^A in ATP5D mRNA recruits YTHDF1 and eRF1 to reduce translation efficiency,^114^ whereas in mitochondrial RNAs, m^1^A impedes effective translation by disrupting Watson‐Crick base pairing (Figure 4F). However, in nuclear mRNAs, m^1^A at the 5′ cap and 5′‐UTR may enhance translation^107^, ^115^ (Figure 4G).

### 2.7 m^7^G

**FIGURE 5:**
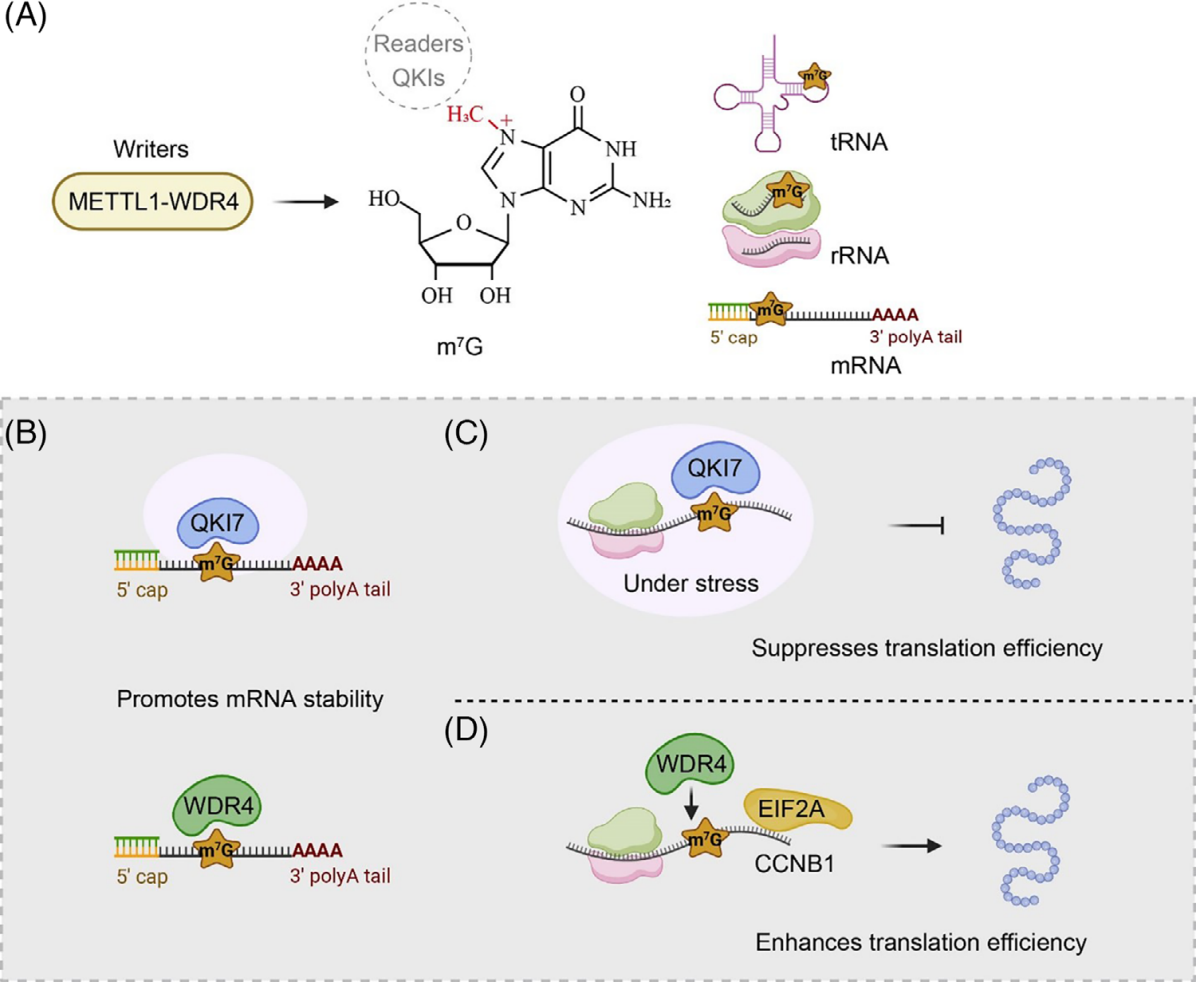
Regulatory factors in modulating *N* ^7^‐methylguanosine (m^7^G). (A) The methyltransferase 1‐WD repeat‐containing protein 4 (METTL1–WD4) complex is implicated in installing m^7^G modification within mRNA regions. Quaking proteins (QKIs) can recognise internal m^7^G modification. (B) Both QKI7 and WDR4 can promote mRNA stability. (C and D) QKI7 suppresses the translation efficiency under stress conditions (C). WDR4 promotes CCNB1 translation by facilitating EIF2A binding to CCNB1 mRNA (D).

The presence of m^7^G was initially recognised as an integral component of the RNA polymerase II transcript cap structure, contributing to various aspects of the mRNA life cycle.^116^, ^117^ Zhang et al. discovered that the proportion of m^7^G in mammalian mRNA was observed to be in the range of .02%–.05%.^118^ In another study, the abundance of m^7^G in mammalian tRNA was reported to be around 60%–85%.^119^ While extensive reviews have thoroughly covered the regulation and functions of m^7^G cap modifications, this review focused on internal m^7^G marks. Chu et al. have made the first discovery of widespread internal m^7^G modifications in eukaryotic mRNAs, utilising a novel Liquid Chromatography‐Electrospray Ionization – Tandem Mass Spectrometry‐based approach.^120^ Their findings indicate that m^7^G levels are responsive to environmental stress, pointing to a novel regulatory function in eukaryotic stress responses.^120^ Recent findings unveiled internal m^7^G modifications on mammalian mRNA molecules, as independently confirmed by distinct research groups.^121^, ^122^, ^123^ To identify these internal m^7^G sites, researchers developed both antibody‐based immunoprecipitation and chemical‐based methods to identify thousands of potential sites within mammalian transcripts.^122^ Importantly, these internal m^7^G marks display dynamic regulation, particularly in response to stress conditions.^122^ Additionally, the methyltransferase 1‐WD repeat‐containing protein 4 (METTL1–WDR4) complex responsible for tRNA m^7^G methylation is also involved in installing m^7^G modifications in mRNA regions bearing tRNA‐like structures^31^, ^123^ (Figure 5A).

Recently, we identified the Quaking proteins (QKIs), comprising of three isoforms (QKI5, QKI6 and QKI7), as the inaugural reader for internal m^7^G modification^123^ (Figure 5A). The results have shown that QKI7 plays a role in regulating the stability and translation efficacy of a specific set of mRNA transcripts with internal m^7^G modifications, especially under conditions of stress^123^ (Figure 5B,C). In the context of hepatocellular carcinoma, WDR4 is associated with RNA stability, tumour progression and treatment resistance^124^ (Figure 5B). Additionally, WDR4 enhances the translation of specific mRNAs, such as CCNB1 (known as CyclinB1), by facilitating binding to translation initiation factors^124^ (Figure 5D). While internal m^7^G modifications are thought to regulate mRNA translation efficiency, a systematic research is required to gain a comprehensive understanding of these mechanisms.

## 3 IMPLICATIONS OF NON‐m^6^A RNA MODIFICATION IN HAEMATOLOGICAL ONCOLOGY

### 3.1 Ac^4^C

The onset, prognosis and progression of cancer disease has been linked to Ac^4^C and its regulators.^41^, ^125^ NAT10 is overexpressed in various tumour types, including hepatocellular carcinoma,^126^ colorectal cancer,^127^ melanoma,^128^ esophageal cancer,^129^ acute myeloid leukaemia (AML), among others. In regards to AML, significantly higher expression levels of NAT10 in the BM samples of 48 newly diagnosed AML patients were observed compared to those of healthy controls.^130^ Moreover, patients with nucleophosmin 1 (NPM1) mutation had higher NAT10 expression levels than those of NPM1 wild‐type patients.^130^ Importantly, NAT10 overexpression showed a direct correlation with poor overall survival and chemotherapy resistance, indicating that NAT10 could potentially be a novel biomarker for AML patients.^130^ Knockdown or pharmacological inhibition of NAT10 dramatically promoted cell cycle arrest in the G1 phase and apoptosis in AML cells, and inhibited proliferation of AML cells, likely through enhancing endoplasmic reticulum stress and activating unfolded protein response pathways, associated with the upregulation of Bax/bak.^131^ In multiple myeloma (MM), Wei et al. confirmed that the translation efficiency of *CEP170* mRNA, which encodes centrosome protein‐170, was increased by the acetylation of its mRNA mediated by NAT10, which could, in turn, enhance the proliferation as well as the chromosomal instability of MM cells.^132^ Moreover, *BCL‐XL* (*BCL2L1*) was identified as a downstream target of NAT10, with overexpression of NAT10 promoting *BCL‐XL* mRNA stability and translation. This process subsequently leads to the inhibition of apoptosis in cells.^133^ NAT10‐mediated activation of the Phosphoinositide 3‐kinases‐Protein Kinase B (PI3K‐AKT) pathway and the subsequent upregulation of CDK4/CDK6 expedites cell proliferation.^133^ The analysis of the The Cancer Genome Atlas (TCGA) dataset by Li et al. revealed that expression level of *THUMPD1*, which encodes a key adaptor in Ac^4^C RNA modifications, was associated with the prognosis in AML patients.^134^

### 3.2 Ψ

Although pseudouridylation is widely studied in various types of solid tumours, research in blood malignancies is relatively limited. A study by Poncet et al. showed that dyskerin is downregulated in B‐chronic lymphocytic leukaemia.^135^ Dyskerin, an enzymatic component of pseudouridylation encoded by the *DKC1* gene, is essential for ribosome biogenesis and for maintaining the stability of the telomerase complex.^136^ In both rRNA and snRNA, dyskerin mediated the conversion of uridine to pseudouridine at particular sites.^137^ The frequency and efficiency with which ribosomes are produced are both affected by uridine modification, which occurs early in the rRNA processing pathway.^136^ In addition, SHQ1, the H/ACA snoRNP assembly component, has been shown to be overexpressed in T‐acute lymphoblastic leukaemia (T‐ALL).^138^ Through a direct binding mechanism, oncogenic NOTCH1 stimulates transcription of the *SHQ1* gene.^138^ Knockout of SHQ1 induced T‐ALL apoptosis in vitro and increased survival time in T‐ALL mice models.^138^ Further RNA‐seq analysis revealed that SHQ1 inactivation can negatively impact RNA splicing, which can in turn result in MYC oncoprotein downregulation.^138^ Overall, these findings raise the possibility that T‐ALL is more reliant on SHQ1‐mediated snRNA pseudouridylation and a fully functioning spliceosome than previously appreciated.

Another Ψ modification enzyme, known as PUS7, has been the subject of research due to its involvement in stress‐induced RNA pseudouridylation. PUS7 plays a crucial role in modifying and activating a novel network of tRFs that target translation initiation.^139^, ^140^ Considering that an increase in protein synthesis can disrupt haematopoietic stem cell (HSC) function and potentially contribute to the development of leukaemia, further studies regarding dysfunction in PUS7 as a contributing factor in haematological malignancies is important. Indeed, Guzzi et al. reported a connection between the loss of PUS7 and chromosome 7 abnormalities, which are commonly observed in myelodysplastic syndromes (MDS). MDS is a group of haematological clonal disorders characterised by dysfunctional haematopoietic stem and progenitor cells (HSPCs) and a high risk of AML development.^139^ HSPCs display heightened sensitivity to changes in pseudouridine levels and protein synthesis.^139^ Notably, the suppression of PUS7 expression results in the diminishment of a specific class of tRNA‐derived small fragments characterised by 5′ terminal oligoguanine (mTOG), leading to an elevation in protein synthesis and significant inhibition of HSPCs differentiation.^139^ Moreover, PUS7‐mediated pseudouridine formation facilitates the binding of mTOG to PABPC1 and the destabilisation of the translation–initiation complex, known as eIF4F. This fine tunes the process of translation and exerts a direct influence on the growth and commitment of embryonic and HSCs.^139^ Guzzi et al. further reported that individuals with high‐risk MDS and secondary AML, characterised by diminished mTOG and PUS7 levels, exhibit a substantial increase in the translation of mTOG‐sensitive transcripts exclusively in BM mononuclear cells.^140^

### 3.3 m^5^C

Cancer researchers have focused mostly on epitranscriptomic RNA m^5^C alteration in malignant solid tumours.^141^ Important impacts of m^5^C writers and erasers in cancer development include proliferation, differentiation, metastasis and therapy response.^142^ NSUN2 is a typical example of m^5^C methylase that has been proven to be involved in a wide variety of pan‐cancer pathways.^143^ Other dysregulations in the expression of m^5^C regulators have been identified towards various types of cancers. In this regard, data analysis from the TCGA database by Ma et al. revealed that the expression of NSUN6 was upregulated in AML samples in comparison to control samples.^144^ Cheng et al. demonstrated that m^5^C methyltransferases such as NSUN3 and DNMT2 can sensitise leukaemia cells to 5‐azacytidine, whereas resistant leukaemia cell lines and primary AML samples have been shown to have higher expression of NSUN1 instead.^145^ Mechanistically, by interacting with the conserved RNA‐binding protein hnRNPK, NSUN3 and DNMT2 can aggregate different transcription factors and remodel the chromatin structure into a 5‐azacitidine‐sensitive formation.^145^ In fact, Cheng et al. stated that hnRNPK acts as a ‘reader’ of written decorations by m^5^C methyltransferases.^145^ NSUN1 can desensitise these cells to 5‐azacitidine via BRD4 and RNA‐polymerase‐II binding to form an insensitive chromatin structure.^145^ Of note, other mechanisms have also been investigated in the drug sensitivity of leukaemia cells to 5‐azacitidine. Schaefer et al. reported that in myeloid leukaemia cells, 5‐azacitidine prevents tRNA methylation at DNMT2 target sites.^146^

In AML, TET2 is a notable player with recurrent mutations, and its insufficiency catalyses the progression of leukaemogenesis, especially when combined with potent oncogenic mutations.^26^ Li et al. demonstrated that TET2, traditionally known for its role in DNA demethylation, also plays a pivotal role in RNA demethylation and specifically m^5^C modification in mRNA targeting.^26^ Beyond its canonical transcriptional gene regulation as a DNA demethylase, TET2 has been identified as an RNA‐binding protein capable of oxidising m^5^C in mRNA as an RNA demethylase, in turn influencing target mRNA stability and expression. In the context of AML, deficiency (downregulation or loss‐of‐function mutations) in TET2 expression/function results in the increased accumulation of m^5^C residues in mRNA.^26^ A critical m^5^C‐modified mRNA target of TET2 is *TSPAN13*, which plays a pivotal role in leukaemia stem cells (LSCs) homing into the BM niche through regulating the CXCR4–CXCL12 axis.^26^ TET2 deficiency enhances the stability and expression of *TSPAN13* via a YBX1‐dependent mechanism, and triggers CXCR4/CXCL12 signalling, promoting LSC homing/proliferation and leukaemogenesis.^26^ This TET2‐mediated post‐transcriptional regulation is central to the TSPAN13/CXCR4 axis, highlighting the significance of the TET2/m^5^C interplay in AML pathogenesis and LSC homing/self‐renewal and suggesting potential therapeutic avenues for TET2‐deficient AML.^26^ These studies collectively emphasise the key regulatory role of m^5^C in haematological malignancies.

### 3.4 A‐to‐I

Recently, ADAR dysfunction has been implicated in cancer development.^73^ For example, ADAR1 is overexpressed in several cancer types, including lung cancer,^147^ liver cancer,^148^ esophageal squamous cell carcinoma,^149^ chronic myelogenous leukaemia.^150^ ADAR1 has been found to enhance the self‐renewal capacity of LSCs by editing let‐7 pri‐microRNA and upregulating LIN28B.^151^ On the other hand, ADAR2 is poorly expressed in certain cancers such as glioblastoma and haematological malignancies, where the reduced amount of ADAR2 editing results in malignant phenotypes.^152^, ^153^, ^154^ For example, Guo et al. reported that in vivo ADAR2 induction can inhibit leukaemogenesis of t(8;21) AML cells through its A‐to‐I editing ability.^153^ Upregulation of ADAR2 via 6‐thioguanine can also reduce ALL cell viability.^154^

Furthermore, Lazzari et al. showed that in serially transplantable patient‐derived xenografts, elevated ADAR1 RNA expression is associated with lower MM patient survival rate, and its knockdown inhibits the regeneration of high‐risk MM.^155^ ADAR and other genes in the 1q^21^ amplicon are linked to tumour growth and/or treatment sensitivity due to the elevation of the expressed genes, which are brought on by the higher gene dosage in MM cells with 1q^21+^.^156^ Moreover, Crews et al. stated that ADAR1 upregulation can lead to increased self‐renewal of malignant MM cells, and the suppression of ADAR1 can prevent MM relapse.^157^ Additionally, in vivo ADAR1 knockout could reduce LSCs.^150^ Single‐sample Gene Set Enrichment Analysis conducted by Zhang et al. unveiled that ADAR expression was negatively associated with immune cell infiltration.^158^ The oncogenic role of ADAR1 was also identified by Shi et al., who confirmed that ADAR1 by regulating Rho GTPase splicing can aid the progression of a rare subtype of non‐Hodgkin's lymphoma, the so‐called ocular adnexal B‐cell lymphoma.^159^

Notably, ADAR level was detected to be increased in Epstein–Barr virus (EBV^+^) paediatric peripheral T‐cell lymphoma patients in comparison to EBV^‐^ patients.^160^ Pecori et al. discovered that ADAR1 can results in the progression of Diffuse Large B‐Cell Lymphoma (DLBCL) via A‐to‐I editing of mitochondrial antiviral signalling (MAVS) transcripts, the downstream adaptor of the RLR.^161^ In fact, increased mRNA levels of MAVS are associated with higher A‐to‐I modification levels, which in turn can initiate tumourigenesis pathways and increase T‐cell exhaustion.^161^ Eventually, recent findings show that A‐to‐I editing can also implement its function by regulating ncRNAs. miRNAs are examples of dsRNA loop structures that are targeted by ADAR1 and in turn can regulate the gene expression of multiple target genes. Zipeto et al. reported that ADAR1 promotes the production of blast crisis in LSCs by inhibiting the expression of miRNAs that silence stem cell regulatory transcripts.^162^ Furthermore, a class of long non‐coding RNAs (lncRNAs), termed snoRNA‐related lncRNAs, which are synthesised through a specific biogenesis pathway of snoRNAs, have also been implicated in haematopoietic malignancies as targets of A‐to‐I editing. For example, Huang et al. reported that LNC‐SNO49AB is upregulated in AML and ALL patients, and can enhance the cell proliferation rates both in vitro and in vivo.^163^ The study demonstrated that LNC‐SNO49AB interacts with ADAR1 and stimulates its homodimerisation, which in turn increases the A‐to‐I editing activity of ADAR1.^163^ Transcriptome profiling data demonstrates obvious parallels between the RNA modification changes induced by LNC‐SNO49AB and those induced by knocking down ADAR1, which are most evident in cell cycle‐related signalling pathways.^163^ Gassner et al. reported that knocking out ADAR results in decreased steady‐state viability of MEC1 cells and increases their susceptibility to treatment with fludarabine and ibrutinib in vitro.^164^ Based on the current evidence, focusing on the targeting of A‐to‐I editing facilitated by ADARs could potentially become a future strategy to enhance treatment efficacy.

### 3.5 N_m_

Zhou et al. demonstrated that LSC gene expression profile and the development stage in AML are strongly correlated with certain rRNA N_m_ patterns.^165^ As a result, LSC self‐renewal is regulated by dynamic N_m_ at particular sites on ribosomal RNAs, thereby changing translational preferences.^165^ FBL serves as a crucial nucleolar protein, actively engaged in the processes of pre‐rRNA methylation and processing. Notably, the methyltransferase domain within FBL stands out as a prime example of a highly conserved protein domain, exhibiting minimal alterations in its amino acid sequence throughout the course of evolution, from Archaea to Eukaryota.^165^ Within the human biological context, the predominant rRNA modification involves N_m_, a modification catalysed by the C/D box snoRNP complex housing the methyltransferase FBL.^165^ A study conducted by Zhou et al. delved into the transcriptome analysis of 90 primary AMLs, revealing a positive correlation between FBL expression and the presence of LSC genes.^165^ These findings strongly suggest a potential link between FBL‐mediated rRNA 2′‐O‐Me and the phenotypic characteristics of LSCs in AML.^165^ Moreover, Pauli et al. found that SNORD42A can increase the proliferation rate of AML cells by 18S‐U116 N_m_ of the leukemic cells.^91^ They showed that reduction in 18S‐U116 N_m_ can be observed by the knockout of SNORD42A and this reduction is associated with a decrease in the translation of ribosomal proteins.^91^ Jiang et al. reported that the mutation in *FTSJ3* can be utilised as a prognosis marker based on B‐cell receptor sequencing data from two different clusters of DLBCL patients.^166^ Begik et al. showed that HENMT1, one of the important 2′‐O‐methyltransferases, is upregulated in AML cells.^167^ Furthermore, Zhou et al. found that C/D box snoRNAs function as paramount determinants of LSC activity and a direct correlation is observed between the global loss of C/D box snoRNAs and a concurrent decline in rRNA N_m_.^168^ This decrement in rRNA N_m_ parallels a diminished self‐renewal capability of LSCs, thereby pointing to its critical function in preserving LSC potential.^168^ Nonetheless, despite the availability of cutting‐edge methods in RNA modification world, precise determination of N_m_ patterns in haematologic malignancies remains to be an unmet goal.

### 3.6 m^1^A

In the context of haematological malignancies, in a study carried out by Esteve‐Puig et al., the presence of m^1^A modifications within the 5′‐UTRs of genes was associated with elevated levels of protein expression.^169^ Notably, their study revealed that the targeted reduction of ALKBH3 through shRNAs in HD‐MY‐Z cells, a cell line associated with Hodgkin lymphoma (HL), resulted in significantly increased protein expression of essential extracellular components, specifically type I α2 and type I α1 collagens, which play pivotal roles within the HL microenvironment.^169^

### 3.7 m^7^G

The eukaryotic translation initiation factor eIF4E is found to be elevated in about 30% of cancer cases, including specific subtypes of AML known as M4/M5 AML. The oncogenic potential of eIF4E arises from its capability to bind to the m^7^G cap on mRNA molecules, selectively promoting eIF4E‐dependent processes such as nuclear mRNA export and translation.^170^ Assouline et al. demonstrated that ribavirin, a broad‐spectrum antiviral drug, acts as a physical mimic of the m^7^G cap, effectively obstructing function of eIF4E.^170^ Orellana et al. have presented compelling evidence suggesting the oncogenic nature of the methyltransferase complex METTL1/WDR4.^171^ Notably, they showed that a deficiency in METTL1 results in a reduction in m^7^G tRNA methylation and a subsequent decrease in overall translation, leading to cell cycle aberrations and the suppression of tumour growth in various xenograft models, including those related to AML.^171^ Their investigation further revealed that the overexpression of METTL1/WDR4 is associated with malignant transformation and the initiation of tumourigenesis.^171^ The underlying mechanism is linked to an elevated level of m^7^G tRNA modification following METTL1 gain‐of‐function, which in turn increases the abundance of specific tRNAs, such as Arg‐TCT‐4‐1. Arg‐TCT4‐1, one of several isodecoder tRNAs responsible for decoding AGA codons (six in humans), leads to heightened translation of mRNA molecules enriched with AGA codons.^171^ This includes transcripts associated with crucial cellular processes like the cell cycle.^171^

## 4 DISCUSSION AND PERSPECTIVE

Accumulating evidence in the realm of non‐m^6^A RNA modifications has provided insights into their regulatory roles in RNA metabolism in haematological malignancies, including RNA stability and translation. Non‐m^6^A RNA modifications intricately govern the functions of diverse RNA types, including mRNA, rRNA and tRNA, which significantly influences the expression of genes associated with haematological malignancies, ultimately contributing to the development of these malignancies. Despite notable advancements, our comprehension of the regulatory mechanisms governing non‐m^6^A mRNA modifications still contains significant gaps. The identities of numerous writers, erasers and readers involved in these modifications remain elusive, hindering a more profound exploration of their functional mechanisms. Additionally, the potential collaborations or competitions among the non‐m^6^A mRNA modifications and m^6^A modification have yet to be systematically investigated. It is noteworthy to emphasise that these findings are crucial for advancing both predictive and therapeutic applications in haematological malignancies. We have summarised that NAT10^130^ and THUMPD1^134^ are potential prognostic predictors for AML. Likewise, a significant association was observed between ADAR1 expression and adverse patient outcomes.^155^ Additionally, a substantial role of ADAR1‐mediated editing of GLI1 as a mechanism influencing both the progression of MM and the emergence of drug resistance was reported.^155^

Better understanding of non‐m^6^A RNA modifications may significantly impact patient care by paving the way for personalised treatment strategies and improved clinical outcomes. However, the development of epitranscriptomic drugs is currently in its early stages. Several ongoing studies aim to decipher the efficacy of these small‐molecule inhibitors on epitranscriptomic transcriptome regulators in haematological malignancies. Crews et al. developed Rebecsinib, an ADAR1p150 inhibitor that reversed splice isoform switching, reducing LSC self‐renewal and extending survival,^172^ offering a promising avenue for AML treatment. Remodelin, previously identified as a potent NAT10 inhibitor related to Ac^4^C RNA modification,^173^ has shown potential in haematological malignancies.^133^ Specifically, it inhibited proliferation and triggered apoptosis in MM and AML cells upon the suppression of NAT10 by Remodelin.^133^ However, a debate exists regarding whether Remodelin interacts directly with the acetyltransferase active site of NAT10^174^ and concerns linger about its potential ‘off‐target’ effects due to its interactions with multiple cellular proteins. In addition, specifically targeting PUSs activity, multiple efforts have been made to pinpoint compounds that could reduce DKC1 activity for potential anticancer applications.^142^ Currently, a clinical trial is underway to assess the potential of Pyrazofurin, a molecule acknowledged for targeting orotodine‐5′‐monophosphate‐decarboxylase and inhibiting DKC1, as a treatment for AML.^175^

**FIGURE 6:**
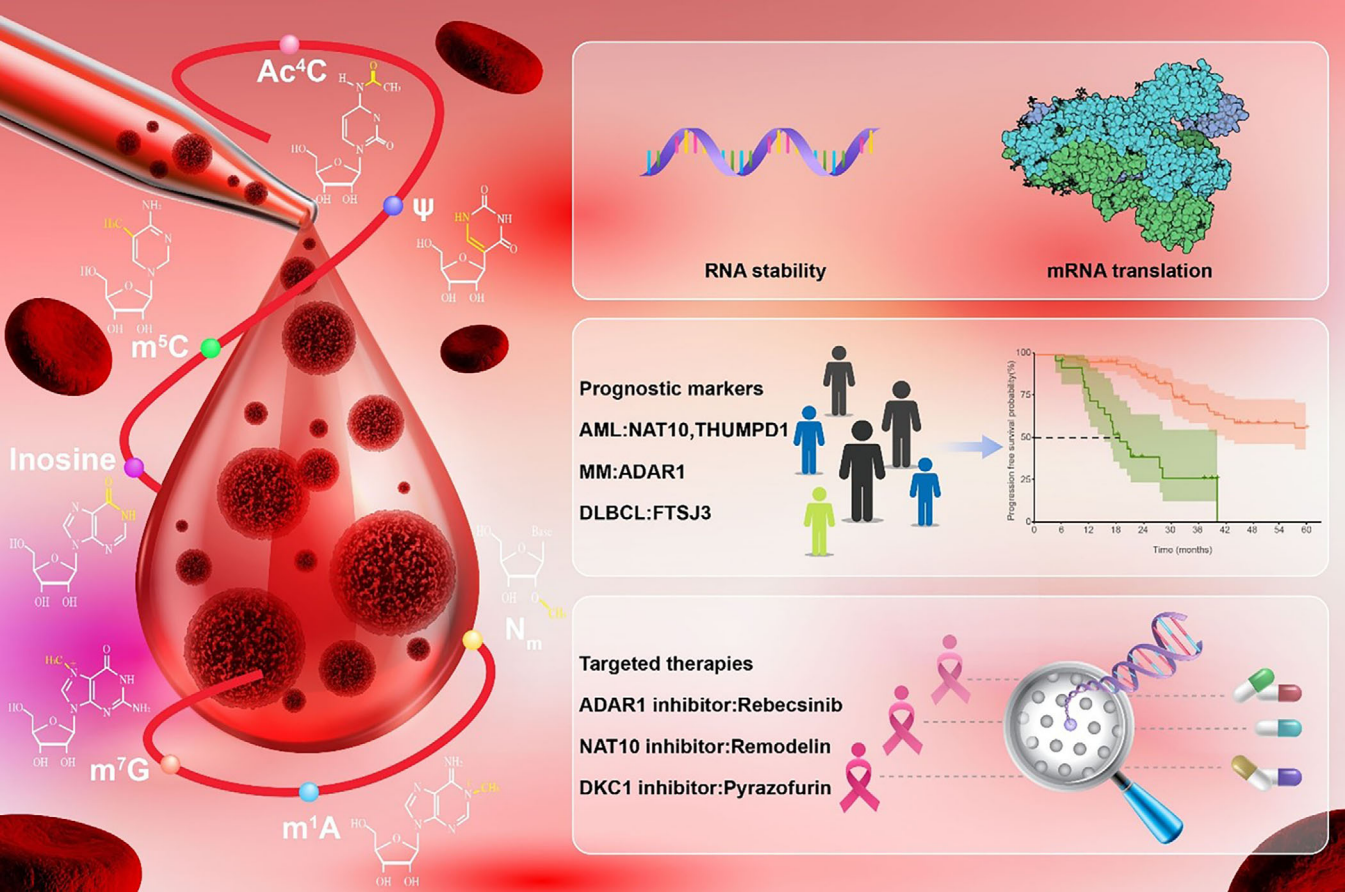
The critical role and emerging understanding of non‐*N* ^6^‐methyladenosine (m^6^A) RNA modifications in haematological malignancies. Non‐m^6^A RNA modifications regulate both RNA stability and translation. The prognostic significance of key enzymes such as N‐acetyltransferase 10 (NAT10) and THUMP domain containing 1 (THUMPD1) in acute myeloid leukaemia (AML), ADAR1 in multiple myeloma (MM) and FTSJ3 in DLBCL are highlighted. Concurrently, emerging therapeutic agents such as Rebecsinib targeting ADAR1, Remodelin against NAT10 and Pyrazofurin as a dyskerin pseudouridine synthase 1 (DKC1) inhibitor are showcased, which are in the early stages of development for treating blood cancers. DLBCL, Diffuse Large B‐Cell Lymphoma.

In summary, epitranscriptomics, especially its association with haematological malignancies, provides valuable insights into RNA metabolism and tumourigenesis pathways. Despite the known importance of RNA modifications such as m^5^C, A‐to‐I RNA editing, N_m_, m^1^A and m^7^G, the pursuit of targeted inhibitors is still in its early stages. The realm of targeting these RNA modifications for therapy is vast, and we eagerly await breakthroughs in developing potent inhibitors (Figure 6).

## 5 CONCLUSION

Studies on epitranscriptomics, the biological importance of RNA modifications, and their regulatory genes are still in a nascent phase. Nevertheless, this dynamic and reversible field continues to draw attention, especially in terms of haematological malignancies. A great deal of advancements has been made in understanding the significance of non‐m^6^A RNA modification regulators, yet the functions of multiple regulators remain unclear. Consequently, to fully understand the epitranscriptomics‐related pathogenic mechanisms of tumours, further research is necessary in the field of epitranscriptomics. Furthermore, no inhibitors, particularly those targeting non‐m^6^A RNA modification regulators, are currently available for clinical use. Thus, further research is necessary to elucidate the precise mechanisms and develop potent inhibitors targeting these modifiers. Such inhibitors can potentially lead to the development of effective new therapies for haematological malignancies. The summary of our review is listed in Table 1.

**TABLE 1:** Summary of epitranscriptomic modulators and their effects in various haematological oncology.

Modification type	Enzymatic category	Regulators	Effects/clinical usage	Cancer types	RNA species	Deregulation	Refs.
Ac^4^C (.01%–.36% in mRNA)	Writer	NAT10	‐ Poor progress‐free survival and overall survival ‐ Its suppression can enhance cell cycle arrest in the G1 phase by targeting CDK2, CDK4, CyclinD1, Cyclin E, Bax/Bcl‐2 axis	AML	mRNA, rRNA, tRNA	Upregulated	^130^, ^131^
			‐ Increase translation efficiency of CEP170	MM	mRNA	Upregulated	^132^
			‐ Increase translation efficiency of BCL‐XL ‐ Activate PI3K–AKT pathway and CDK4/CDK6	MM	mRNA	Upregulated	^133^
			‐ THUMPD1 ‐ Potential predictor marker	AML	mRNA	–	^134^
Ψ (.2%–.6% in mRNA and 1.4% in rRNA)	Writer	Dyskerin	–	B‐CLL	mRNA	Downregulated	^135^
		SHQ1 (H/ACA snoRNP)	‐ Stimulated by NOTCH1	T‐ALL	mRNA	Upregulated	^138^
		PUS7	‐ Facilitates the binding of mTOG to PABPC1	MDS, AML	tRNA	Downregulated	^139^
m^5^C (.02%–.09% in mRNA)	Writer	NSUN6	–	AML	N/A	Upregulated	^144^
		NSUN3 and DNMT2	‐ Sensitise leukaemia cells to 5‐azacytidine	AML	tRNA, mRNA	–	^145^
		NSUN1	–	AML/MDS	mRNA	Upregulated	^145^
	Eraser	TET2	‐ Decreased expression of TET2 augments the capacity for self‐renewal among LSCs	AML	tRNA, mRNA	Mutated	^26^
A‐to‐I (N/A)	Writer	ADAR2	‐ Guo M, Chan THM, Zhou Q, et al.	AML	N/A	–	^153^
			‐ Upregulated ADAR2 can reduce cell viability	ALL	mRNA	–	^154^
		ADAR1	‐ Elevated ADAR1 expression is associated with lower survival rate	MM	mRNA	Upregulated	^155^
			‐ Regulate the Rho GTPase splicing	Non‐Hodgkin's lymphoma	mRNA	–	^159^
			‐ Increase A‐to‐I editing of MAVS transcripts ‐ Increase T‐cell exhaustion	DLBCL	mRNA	Upregulated	^161^
			‐ LNC‐SNO49AB is upregulated by increased level of ADAR1	AML/ALL	rRNA	Upregulated	^163^
		ADAR	‐ Increase self‐renewal of malignant	MM	mRNA	Upregulated	^157^
			‐ ADAR expression is negatively associated with immune cell infiltration	MM	mRNA	–	^158^
			–	EBV^+^ paediatric peripheral; T‐cell lymphoma	N/A	Upregulated	^160^
N_m_ (.012%–.15% in mRNA)	Writer	FBL	‐ Different rRNA 2ʹ‐O‐methylation patterns related to AML‐LSC self‐renewal	AML	rRNA	–	^165^
		SNORD42A	‐ Increase 18S‐U116 2′‐O‐methylation	AML	rRNA	Upregulated	^91^
		FTSJ3	‐ Act as a prognosis marker	DLBCL	rRNA	–	^166^
		HENMT1	–	AML	piRNA	Upregulated	^167^
m^1^A (.015%–.054% in cells and .16% in tissues)	Eraser	ALKBH3	‐ Increase the expression of collagens	HL	mRNA	Downregulated	^169^
m^7^G (.02%–.05% in mRNA)	Writer	METTL1	‐ Malignant transformation	AML	tRNA	Upregulated	^171^

*Note*: This table provides an overview of epitranscriptomic modulators and their associated effects, clinical relevance, cancer types and deregulation status.

Abbreviations: A‐to‐I, adenosine to inosine; Ac^4^C, *N* ^4^‐acetylcytidine; ALL, acute lymphoblastic leukaemia; AML, acute myeloid leukaemia; B‐CLL, B‐chronic lymphocytic leukaemia; DLBCL, Diffuse Large B‐Cell Lymphoma; EBV, Epstein–Barr virus; FBL, fibrillarin; HL, Hodgkin lymphoma; LSC, leukaemia stem cell; m^1^A, *N* ^1^‐methyladenosine; m^5^C, 5‐methylcytosine; m^7^G, *N* ^7^‐methylguanosine; MAVS, mitochondrial antiviral signalling; MDS, myelodysplastic syndrome; METTL1, methyltransferase 1; MM, multiple myeloma; mTOG, 5′ terminal oligoguanine; N_m_, 2′‐O‐methylation; NAT10, N‐acetyltransferase 10; piRNA, piwi‐interacting RNA; PI3K‐AKT, Phosphoinositide 3‐kinases‐Protein Kinase B; PUS, pseudouridine synthase; snoRNP, small nucleolar ribonucleoprotein; T‐ALL, T‐acute lymphoblastic leukaemia; TET, ten‐eleven translocation; THUMPD1, THUMP domain containing 1; Ψ, pseudouridylation.

## AUTHOR CONTRIBUTIONS

Meiling Chen, Yuanzhong Chen, Kitty Wang, Xiaolan Deng and Jianjun Chen wrote the manuscript and created the figures.

## CONFLICT OF INTEREST STATEMENT

The authors declare they have no conflicts of interest.

## ETHICS STATEMENT

Not applicable.

## Data Availability

Further information may be directed to and will be fulfilled by the lead contact, Jianjun Chen (jianchen@coh.org).
